# The Role of Informal Mentors in Promoting Resilience of Latino/a Late Adolescents and Emerging Adults

**DOI:** 10.3390/bs16060910

**Published:** 2026-06-03

**Authors:** Gabriel P. Kuperminc, Maria Alejandra Arce, Roushanac Partovi, Kathleen M. Roche

**Affiliations:** 1 Department of Psychology, Georgia State University, Atlanta, GA 30302, USA; 2 Department of Psychology, University of California, Riverside, Riverside, CA 92521, USA; malejandra.arce@ucr.edu; 3 Department of Social Medicine, Population and Public Health, University of California, Irvine, CA 92617, USA; rpartovi@uci.edu; 4 Department of Prevention and Community Health, Milken Institute School of Public Health, The George Washington University, Washington, DC 20052, USA; kroche@email.gwu.edu

**Keywords:** informal mentoring, resilience, Latino/a youth development, discrimination, behavioral health, internalizing problems, externalizing problems

## Abstract

This study examined the compensatory and risk-protective roles of informal mentoring in the longitudinal associations between discrimination and behavioral health problems among Latino/a adolescents and emerging adults. The study addressed the limited research on mentoring among Latino/a youth, particularly with regard to behavioral health, and considered both the presence of and relationship quality with mentors as well as gender differences (girls vs. boys). Latent growth curve analysis was used to investigate trajectories of internalizing and externalizing problems across 11 observations spanning 7 years. Mentor presence assessed at Wave 9 when youth (*N* = 544) were approximately 15–18 years old had an association with internalizing problems consistent with the compensatory model, in which a resilience factor offsets the harmful effects of a risk factor, for girls. Among girls with a mentor, relationship quality buffered associations between discrimination and internalizing problems, consistent with the risk protective model. Among boys who reported having a mentor, relationship quality had an association with internalizing problems consistent with the compensatory model. For both girls and boys with mentors, relationship quality buffered associations between discrimination and externalizing problems, consistent with the risk protective model. Whereas discrimination consistently shows harmful effects on Latino/a adolescents’ emotional and behavioral adjustment, a resilience perspective underscores the reality that, with adequate support, most youth are able to overcome those risks. The current study fills gaps in the literature by examining how informal mentoring can foster resilience to such harmful effects, and highlights directions for future research and practice aimed at enhancing the well-being of this large and fast-growing population.

## 1. Introduction

Mentoring has garnered increased research attention for its role in fostering positive development and health among children and adolescents. Over the past three decades, research has consistently shown that participation in programs in which youth are matched with a caring adult or older peer is linked to improvements across a range of academic, social, cognitive, and psychological developmental outcomes (DuBois et al., 2011; Raposa et al., 2019). Research on informal mentoring relationships that form organically between youth and adults in their social network (e.g., extended family members, teachers, or coaches) has found that the presence of informal (sometimes referred to as a “natural”) mentors in young people’s lives contributes to improvements in academic, social and vocational development, with stronger effects in these domains evident when youth experience high relationship quality with mentors in terms of relatedness, social support, and autonomy support (Van Dam et al., 2018).

Much of the research on informal mentoring has focused on youth of color, primarily Black or African American youth, or has examined diverse samples without considering the unique characteristics or needs of differing groups. Groups and individuals differ in rates of behavioral health challenges, exposure to discrimination, and the ways in which relational processes like mentoring can help youth navigate challenges in their everyday lives. De Los Reyes et al. (2022) argued that Latino/a^1^ youth have unique needs and are vulnerable to poor developmental outcomes because of systemic and interpersonal racism. Despite these risks, most Latino/a youth manage to overcome adversities in their lives (Kuperminc et al., 2009). Using a resilience perspective, the current study examines the role of informal mentors in disrupting longitudinal associations between late adolescents’ experience of everyday discrimination and their emotional and behavioral health.

Research suggests that the experiences of immigrant-origin children and youth (IOC&Y), and Latino communities specifically, have implications for understanding resilience processes in development (Kuperminc et al., 2009; Suárez-Orozco et al., 2018). In their integrative model of risk and resilience, Suárez-Orozco et al. (2018) highlight that societal contexts of racism and negative attitudes toward immigration shape how IOC&Y approach normative developmental and acculturative tasks. Their model differentiates the experiences of U.S.-born youth from those who immigrated to the U.S., noting, for example, that while both groups are tasked with acquiring bi-cultural competencies, a dual frame of reference may allow immigrants to see how their current living conditions have improved post-migration. Such a perspective can support optimism, motivate individuals to make the best of their situation, and shield immigrants from effects of discriminatory experiences (e.g., Arce et al., 2024). Compared to their U.S.-born peers, immigrants may face challenges in navigating acculturative and developmental transitions due to needing to learn a new language and adjust to a new and often hostile receiving context (e.g., Schwartz et al., 2014).

### 1.1. Role of Relationships in Promoting Resilience

Research interest in how relationships with non-parental adults can foster successful outcomes among children and adolescents facing risks to their development dates to the 1950s when Emmy Werner’s pioneering studies of the psychosocial resilience of participants who were exposed to multiple stressors, including poverty, perinatal stress, parental divorce, parental alcoholism, and/or mental illness. Affectionate bonds with caregivers outside the immediate family, such as neighbors, relatives, teachers and others, were consistently cited as present in the lives of participants who were identified as resilient from childhood to adulthood (Werner, 2005). As the field of resilience research has continued to mature, researchers have consistently placed such close relationships on the “short list” of potential promotive factors (Masten & Obradović, 2006). Similarly, positive youth development (PYD) research has consistently found relationships with important adults to be among the most important assets in adolescents’ lives for promoting thriving or high levels of PYD and low levels of risk behaviors (Bowers et al., 2015).

Current definitions of resilience emphasize the capacity of developmental systems to adapt successfully to challenges that threaten functioning, survival, or future development (Masten & Barnes, 2018). This definition emphasizes that resilience does not reside within individuals, but rather, depends on individuals’ connections to other people and systems with whom they come into contact. Variable-centered methods of studying resilience test for associations between measures of risk or adversity and adaptative outcomes, alongside one or more potential explanatory variables hypothesized to mediate, counteract or moderate the effects of risk or adversity on adaptive outcomes (Masten & Barnes, 2018). These explanatory variables include internal assets (e.g., self-regulatory competence, internal motivation) and external resources (e.g., familial and extrafamilial support systems). Two primary models include the compensatory model and the risk-protective model (Zimmerman et al., 2013). In the compensatory model, resilience factors directly counteract exposure to risk and are assessed by examining the main effects of the promotive factor in a regression equation. In the risk-protective model, resilience factors mitigate the negative effects of risks and can be assessed by examining multiplicative risk X resilience factor interaction terms in a regression equation.

### 1.2. Discrimination and Behavioral Health Among Latino/a Youth

Discrimination has been consistently associated with worse behavioral health outcomes among U.S. Latinos (Andrade et al., 2021; Assari et al., 2017), with some evidence that Latino/a Americans are more negatively affected than other ethnic/minority groups (Andrade et al., 2021). Research with Latino/a adolescents has found cumulative and lasting effects of discrimination on multiple developmental and behavioral health outcomes, including higher levels of depression and anxiety (Andrade et al., 2021; Stein et al., 2019) and lower levels of prosocial behaviors (Partovi et al., 2025). Using data from the first two waves of the current study, Bennett et al. (2020) found that discrimination experienced at school by teachers and/or other adults was directly associated with more internalizing and externalizing problems over a 6-month period. In a follow-up study spanning the first five waves of data, Bennett et al. (2024) found indirect effects of peer discrimination on externalizing but not internalizing problems via affiliation with deviant peers.

Effects of discrimination can vary depending on the source (e.g., peers, adults) or the setting (e.g., school, community) in which it takes place. Discrimination can be defined as “dominant group members’ actions that have a differential and negative effect on subordinate racial ⁄ ethnic groups” (Seaton et al., 2009, p. 407), and operationalized in terms of daily hassles and micro-aggressions. Using this approach, research has shown associations between everyday discrimination experiences and depression as much as three years later (Assari et al., 2017; Choi et al., 2022). Despite the risks and consequences of discrimination for Latino/a youth behavioral health, there is a fundamental human capacity for resilience that is not an extraordinary trait (Masten, 2001) but rather a process that can be fostered through adequate supports and resources, such as the presence of supportive mentors (Sánchez et al., 2017; Zimmerman et al., 2013).

### 1.3. Mentoring and Promotion of Behavioral Health Among Latino Youth

Developmental science has long recognized that relationships formed with non-parental adults take on increased importance in adolescence, as young people seek behavioral and psychological autonomy from parents (Hartup, 1989; Oudekerk et al., 2015). Such relationships often take the form of mentorship, either through formal programs or interaction with adults that young people encounter in everyday life (Bowers et al., 2015). Findings from Van Dam et al.’s (2018) meta-analyses on the effects of the presence of informal mentors and of relationship quality with mentors on youth adjustment across multiple domains showed larger overall effect sizes for relationship quality, *r* = 0.21, than for mentor presence, *r* = 0.11. Additionally, effects of mentoring on academic/vocational and positive socioemotional development were larger than those for psychosocial problems. It should be noted that studies rarely assess mentor presence and relationship quality simultaneously; studies typically compare youth with vs. without a mentor (mentor presence) or examine the role of relationship quality within samples of youth with one or more identified mentors. Thus, the weaker associations found for mentor presence likely reflect lack of attention to variability in relationship quality.

Van Dam et al. found no differences in the effect sizes for youth characteristics, including gender and ethnicity, risk status, or most mentor characteristics. As an exception, having a mentor who was a helping professional was associated with more positive youth outcomes. Because research on informal mentoring has largely failed to consider variations in processes or outcomes by race or ethnicity (De Los Reyes et al., 2022), however, the meta-analytic analysis may not be sensitive to potentially important ethnic variations.

Much of the research on informal mentoring has been framed within a resilience perspective, often targeting specific minoritized groups or sampling predominantly ethnic minority groups presumed to be exposed to environments that increase their risk for negative developmental outcomes. The proportion of youth reporting having a mentor in these studies has ranged from 35% to more than 70% of youth identifying at least one mentor in their lives, with much of this variation attributable to how mentors were defined (e.g., whether family members and those close in age to the youth were excluded from the definition). Studies have found evidence for both the risk-protective and compensatory models.

Consistent with the compensatory model, in two of the first studies of informal mentoring Rhodes and colleagues (Rhodes et al., 1992, 1994), found cross-sectional associations of mentor presence with fewer depression symptoms among low-income adolescent mothers. With regard to the risk-protective model, Cooper et al.’s (2013) cross-sectional study of a Black adolescent sample (*N* = 4256) found that mentor presence buffered associations of discrimination with school suspensions for both girls and boys, and with school engagement for boys only. Similarly, Zimmerman et al.’s (2002) cross-sectional study found mentor presence interacted with measures of risk, including friends’ problem behavior and norms supporting problem behavior, to predict fewer youth problem behaviors. Longitudinal studies of mentor presence have more commonly found evidence for the compensatory model (e.g., DuBois et al., 2011; Hurd & Zimmerman, 2010b, 2010a). An exception is Hurd and Zimmerman’s (2010a, 2010b) study of Black adolescent mothers, which found evidence for both the compensatory and risk-protective models in that mentor presence was linked to reductions in depressive symptoms over time and buffered effects of perceived stress on anxiety symptoms.

Compared to studies examining mentor presence, relatively few studies have considered the quality of informal mentoring relationships as a resilience factor. However, studies of broader constructs, such as social support (Cano et al., 2020; Sabina et al., 2022) offer evidence for considering both the compensatory and risk-protective models for the role of relationship quality in informal mentoring. In a recent cross-sectional study, mentor support buffered the association between school-based discrimination and psychological well-being for African America boys (Khahra et al., 2024). Kogan et al.’s (2011) three-wave study found that Black emerging adults’ (*N* = 345) relationships with informal mentors, characterized by instrumental and emotional support, were linked longitudinally to lower levels of anger, rule breaking, and aggression.

In one of the few studies of informal mentoring that addressed effects of discrimination on Latino/a youths’ psychological well-being, Sánchez et al. (2017) found complex links between discrimination, mentoring, and youths’ sense of coping efficacy. Specifically, higher levels of discrimination reported in grade 9 were associated with lower coping efficacy and lower relationship quality with mentors. However, one aspect of the mentoring relationship—higher instrumental mentoring quality in 9th grade—contributed to higher coping efficacy a year later.

Most studies of informal mentoring for Latino/a youth have focused on academic outcomes and cultural identity development (De Los Reyes et al., 2022). Cross-sectional study results have shown that mentor presence is associated with fewer school absences and more positive attitudes toward education (Anderson et al., 2019; Sánchez et al., 2008). Studies that examine mentor–mentee relationship quality offer a nuanced picture of how mentoring might contribute to resilience processes. For example, a positive relationship quality appears to indirectly increase youth’s positive attitudes toward education through growth in ethnic identity (Sánchez et al., 2020) and intrinsic motivation (Anderson et al., 2019). Liao and Sánchez (2019) used cluster analysis to identify groups of Latino/a youth whose mentor relationships were characterized as either (a) less close and less growth oriented or (b) close and more growth oriented. The latter group reported more positive attitudes toward education and grade point average than both the former group and a group of youth who did not have a mentor. Furthermore, youth whose mentor relationships were less close and less growth oriented did not differ on most measures from youth without mentors, but had lower grade point averages, suggesting that variation in the quality of relationships with mentors might help explain the weaker associations typically found for mentor presence. Thus, while research specifically focused on Latino/a adolescent samples finds positive effects of informal mentoring, it is unclear whether those effects translate to behavioral health.

### 1.4. Role of Gender

It is important to consider the role of gender, given past research that has established both similarities and differences in the extent and trajectories of emotional and behavioral problems and in the processes that contribute to them (e.g., Leadbeater et al., 1999). Gendered socialization patterns can set girls and boys on different developmental trajectories (Suárez-Orozco et al., 2018). For example, comparatively greater restrictions on social activities may place girls at greater risk than boys of internalizing problems, such as depression and anxiety. Boys, on the other hand, may face greater exposure to harmful stereotypes and lead to risky behaviors, such as delinquency (Suárez-Orozco et al., 2018). Gender differences have been found in exposure to discrimination, its association with behavioral health, and associated resilience resources (e.g., Cooper et al., 2013). While some studies have found that boys experience more discrimination and are more susceptible to its psychological effects than girls (Assari et al., 2017), other studies have found the opposite (e.g., Lorenzo-Blanco et al., 2011). A longitudinal study spanning early adolescence to young adulthood by Delgado et al. (2019) found that friendship intimacy buffered the association between discrimination and depressive symptoms for Mexican-origin girls, but not boys. With regard to mentoring processes, Liang et al. (2014) argue that although both boys and girls can benefit from instrumental and emotional support in mentoring relationships, mentoring processes might differ by gender due to differences in help-seeking behaviors and patterns of relationship development.

### 1.5. Current Study

In sum, although relatively little research has focused specifically on Latino/a adolescents, there is evidence that informal mentoring can contribute to resilience in the psychosocial development of diverse samples. Studies that examine the quality of relationships have tended to find stronger effects, compared with studies that examine only the presence of a mentor, linking informal mentoring with positive outcomes. In this study, we examine both mentor presence and (for youth who identify a mentor) the quality of the mentor–mentee relationship. Because studies generally have not simultaneously examined mentor presence and relationship quality among youth who report having a mentor, studies that focus only on mentor presence conflate the effects of variations in relationship quality, while studies that focus on relationship quality alone are unable to consider how developmental processes might unfold in the absence of mentoring.

The current study addresses the limited research on how informal mentors may contribute to behavioral health among Latino/a youth. We first conducted descriptive analyses, taking into account potential differences by gender, age, immigration status, and maternal education, to examine longitudinal trajectories of internalizing and externalizing problems over a seven-year period spanning middle school to early adulthood. Although mentoring measures were not introduced into the study until youth were in the middle and late adolescent years (i.e., 15–18 years), we capitalize on examining how informal mentoring might contribute to altering the long-term trajectories of internalizing and externalizing problems. We simultaneously examined youth reports of having a mentor alongside ratings of the quality of relationships for youth who identified mentors. Using a resilience framework, we examined the role of informal mentoring in the associations of everyday discrimination with internalizing and externalizing problems. Specifically, we considered informal mentoring as a potential compensatory factor, offsetting associations of discrimination with higher levels of emotional and behavioral problems via main effects, and, as a potential risk-protective factor, buffering the longitudinal associations between discrimination and youths’ internalizing and externalizing problems.

Regarding discrimination, we tested the following hypotheses:

**H1a.**  *Higher levels of discrimination will be associated with more internalizing problems.*

**H1b.**  *Higher levels of discrimination will be associated with more externalizing problems.*

We expect that associations between mentoring variables and both internalizing and externalizing problems will be consistent with the compensatory model of resilience. Specifically, we hypothesize that:

**H2a.**  *Accounting for a positive association between discrimination and internalizing problems, mentor presence will be associated with fewer internalizing problems at W9 and declining internalizing problems through W10 and W11.*

**H2b.**  *Accounting for a positive association between discrimination and externalizing problems, mentor presence will be associated with fewer externalizing problems at W9 and declining externalizing problems through W10 and W11.*

**H3a.**  *Accounting for a positive association between discrimination and internalizing problems, among youth with a mentor, a higher quality mentor relationship at W9 will be associated with fewer internalizing problems and declining internalizing problems through W10 and W11.*

**H3b.**  *Accounting for a positive association between discrimination and externalizing problems, among youth with a mentor, a higher quality mentor relationship at W9 will be associated with fewer externalizing problems and declining externalizing problems through W10 and W11.*

With regard to the risk-protective model of resilience, we hypothesize that mentoring variables will moderate the association between discrimination and slopes for both internalizing and externalizing problems. Specifically, we expect that:

**H4a.**  *Mentor presence at W9 will moderate the association between discrimination and the slope of internalizing problems, such that less increase (or greater decline) in internalizing symptoms through W1 and 11 will be found for youth with a mentor.*

**H4b.**  *Mentor presence at W9 will moderate the association between discrimination and the slope of internalizing problems, such that less increase (or greater decline) in externalizing symptoms through W1 and 11 will be found for youth with a mentor.*

**H5a.**  *Among youth with a mentor, more positive mentor relationship quality at W9 will moderate the association between discrimination and the slope of internalizing problems, such that less increase (or greater decline) in internalizing symptoms through W1 and 11 will be found for youth reporting higher relationship quality.*

**H5b.**  *Among youth with a mentor, more positive mentor relationship quality at W9 will moderate the association between discrimination and the slope of internalizing problems, such that less increase (or greater decline) in externalizing symptoms through W1 and 11 will be found for youth reporting higher relationship quality.*

## 2. Materials and Methods

### 2.1. Study Participants and Procedures

This study used data for 547 Latino/a youth-mother dyads in an ongoing longitudinal study examining how family, community, and societal factors shape youth adjustment from middle school through early adulthood. Youth were initially sampled from 14 middle schools in an emerging immigrant community in metropolitan Atlanta, GA school district. The 14 participating schools, selected to capture the varied range of family, school, and community characteristics, were clustered by the proportion of Latino/a students: low (<13%; *N* = 4); moderate (18–25%, *N* = 6); and high (>40%, *N* = 4). Using 2017–2018 school enrollment lists, 1105 students were randomly selected from grades 6 through 8 for eligibility screening. Youth were eligible if they self-identified as Latina/o or another pan-ethnic or national origin term (e.g., Hispanic, Mexican), read and wrote in Spanish and/or English, and did not have an individualized education plan indicating significant emotional or cognitive developmental disabilities that would prevent them from independently completing self-administered surveys. Youth were also excluded if they already had a sibling enrolled in the study. Recruitment materials were provided in English and Spanish. Parents provided oral or written permission and youth provided written assent. Of the 1105 adolescents selected for screening, 246 parents were unreachable, and 20 students were determined to be ineligible. The response rate was 65.2% (547/839) among eligible youth whose parents were reachable for permission, and 95.3% (547/574) among reachable youth who provided assent. Additional details on sampling and procedures can be found elsewhere (Roche et al., 2020).

Baseline surveys for most youth (77.1%) were completed in school from February through June 2018 (the “main” sample). Due to an unexpected request from the school district to stop in-school data collection by May 2018, data collection transitioned to online administration, with 22.9% of students completing baseline surveys from September 2018 through January 2019 (the “lagged” sample). Youth surveys were self-administered online and recorded using the Qualtrics survey platform. Youth received incentive gift cards of $20 at the baseline assessment, with incentives increasing to $75 in later waves. Investigators obtained a Certificate of Confidentiality from the National Institutes of Health and institutional review board approval (IRB# 121613).

The current study used 11 waves of data spanning seven years. The final analytic sample included 544 participants, as *N* = 3 boys had incomplete data on internalizing and externalizing problems across all waves. Data were administered approximately six months apart for the first eight waves (W1–W8; Spring 2018 through Fall 2021, and one year apart in subsequent waves (W9–W11, Fall 2022 through Fall 2024). At W1 90.7% of youth were in middle school (grades 6–8; the lagged sample completed baseline surveys the following academic year, when a small proportion were in the 9th grade. At baseline, youth were, on average, 13.31 (SD = 0.97) years old, and 55.4% were girls (See Table 1). Many identified solely as Latin American (42.1%), with the remainder identifying with an additional racial group, including White (31%), Black (5.1%), Multiracial (6.4%), Native American or Indigenous (2.8%), or another unspecified racial/ethnic background (10.9%). Most (89.8%) were born in the U.S. Mothers’ educational attainment levels included less than high school completion (39.3%), to high school graduate (23%), some college (8.6%), and college completion or higher (29%).

### 2.2. Measures

#### 2.2.1. Internalizing and Externalizing Problems

For youth aged 18 and younger, youth responses to the Youth Self Report (YSR; Achenbach & Edelbrock, 1991) were used to assess internalizing (29-item) and externalizing problems (30-item); and for youth aged 19 and older, youth responses to the Adult Self Report (ASR; Achenbach & Rescorla, 2003) were used to assess internalizing (37-item) and externalizing (33-item) problems. Data were collected from W1–W11 surveys. Response options include 0 = not true, 1 = somewhat or sometimes true, and 2 = very true or often true. Open ended items were omitted. The internalizing total score is the sum of items assessing: (1) anxious/depressed (12 items YSR and 18 items ASR; e.g., “I am nervous or tense”); (2) withdrawn/depressed (8 items YSR and 9 items ASR; e.g., “I would rather be alone than with others”); and (3) somatic complaints (9 items YSR and 10 items ASR; e.g., “I feel dizzy or lightheaded”). The externalizing total score is the sum of items assessing: (1) rule-breaking behaviors (13 items YSR and 12 items ASR; e.g., “I lie or cheat”); (2) aggressive behavior (17 items YSR and 15 items ASR; e.g., “I destroy my own things”); and (3) intrusive behaviors (6 items ASR; e.g., “I try to get a lot of attention”). Internal consistency estimates were α = 0.80 or higher at each wave of the study. T-scores were calculated based on adolescent gender norms for the total scores on internalizing and externalizing problems. T-scores from the youth and adult versions were then combined to create a single score for internalizing problems and a single score for externalizing problems.

#### 2.2.2. Presence of an Informal Mentor

In W9, when mentoring was first assessed, the presence of an informal mentor was measured with the following prompt adapted from prior research with Latino/a adolescents (Anderson et al., 2019; Rhodes et al., 1994; Sánchez et al., 2008): “Is there an adult in your life who is older and more experienced than you and who you go to for support and guidance? This person is not a parent or the person who raised you or a boy/girlfriend. This is someone who: (a) you can count on to be there for you, (b) believes in you and cares deeply about you, (c) inspires you to do your best, (d) has really influenced what you do and the choices you make.” Response options were modified from listing mentors to indicating the presence of any mentor (0 = no and 1 = yes).

#### 2.2.3. Mentoring Relationship Quality

For youth who reported more than one informal mentor, instructions prompted them to think about the person they could count on the most. Youth then completed a 10-item relational mentoring support subscale (e.g., “The adult I’m close to really cares about me”) and a 6-item instrumental mentoring support subscale (“I talk to the adult I’m close to when I have problems or worries”) taken from the Youth Mentoring Survey (Harris & Nakkula, 2018). Response options ranged from 1 = not at all to 4 = very much. The measure has good psychometric properties and has shown good internal reliability in a past study with Latino/a youth (Anderson et al., 2019). Because the two subscales were strongly correlated (r = 0.73), we averaged the items to create an overall relationship quality measure (α = 0.93).

#### 2.2.4. Everyday Discrimination

The Daily Life Experiences (DLE) subscale of the Racism and Life Experiences Scale (Seaton et al., 2009) was used at W9 to assess past year experiences with micro-aggressions because of race/ethnicity. This measure was added to the study at W9 to replace school-based discrimination measures that were not relevant for youth who no longer attended school. A prompt modified to read, “How often in the past year have the following things happened to you because you are Latino/a?” was followed by 18 items describing experiences of daily discrimination (e.g., “Being insulted, called a name or harassed”). Responses were given on a 6-point Likert scale from 0 = never to 5 = once a week or more. Internal consistency was α = 0.95.

#### 2.2.5. Covariates

The following were included as W1 covariates: adolescent age in years, maternal education, and immigration status. Maternal education was assessed on a 6-point scale ranging from less than high school to graduate or professional school. Immigration status was assessed with a dummy coded variable indicating whether the youth was born in the U.S. All analyses examined gender differences (girls = 0 and boys = 1), using gender reported at baseline. One youth who identified as “Latinx” at baseline was recoded using gender reported in school enrollment records to allow inclusion in models examining gender differences.

### 2.3. Analysis Plan

Preliminary analyses examined descriptive statistics and bivariate correlations for all study variables. For longitudinal structural equation models, we used Mplus 8.0 (Muthén & Muthén, 1998–2017). Following (Hu & Bentler, 1999) we evaluated models using a combination of incremental (CFI ≥ 0.95) and absolute fit indices (RMSEA ≤ 0.06, and SRMR < 0.08) to indicate good fit. Noting that such stringent guidelines can lead to rejecting well-specified models (Marsh et al., 2004), we also considered models with CFI > 0.90, RMSEA < 0.08, and SRMR < 0.10 as demonstrating adequate fit. We first examined latent growth curve models for internalizing and externalizing problems across W1–W11, and used multiple-group models to examine gender differences in levels and patterns of change, estimating linear and quadratic slopes.

We next tested explanatory models in which the intercepts and slopes of internalizing and externalizing problems were regressed on (a) discrimination experiences, (b) the presence of an informal mentor, (c) the overall quality of the mentor–mentee relationship for youth who reported having a mentor, and (d) the interactions of discrimination with the two mentoring variables. Interaction terms were computed by centering discrimination and mentor relationship quality to a mean of 0 before multiplying them together. Consistent with the resilience approach, we examined discrimination as a risk factor increasing the likelihood of internalizing and externalizing problems, and mentoring variables as resilience factors via the compensatory and protective factor models (Zimmerman et al., 2013). To examine concurrent associations of mentoring variables and discrimination with internalizing and externalizing problems, we set the intercepts of these models at W9. We also examined associations of mentoring variables, discrimination and their interactions with the longitudinal slopes of internalizing and externalizing problems, focusing on predicted changes in the trajectories in subsequent waves of the study. We interpreted main effects of mentoring variables (presence and quality of relationship) on internalizing and externalizing problems as reflecting a compensatory process; that is, counteracting exposure to risk through an opposite, direct, and independent effect. We interpreted interactions of mentoring variables X discrimination as reflecting a protective process; that is, moderating or buffering the negative effects of risks.

### 2.4. Missing Data

The majority (76%) of adolescents completed at least seven of the 11 waves of data, and two-thirds (62%) completed W9, when we introduced measures of mentoring. To assess for potential bias due to survey non-response, we examined patterns of missing surveys across all 11 waves. Patterns of missingness represented by less than or equal to 5% were considered “inconsequential” and treated as missing completely at random (MCAR; Schafer, 1999). For 93.2% of the sample, there was little evidence of systematic patterns of missingness, with fewer than 5% of participants exhibiting similar non-response patterns. The remaining 6.8% of adolescents shared a similar pattern of non-response, specifically dropping out at W2 when data collection shifted from in-school to online. A higher proportion of boys (10.7%) compared to girls (3.6%) were lost to attrition at W2 [χ^2^ (1) = 10.58, *p* < 0.01]. However, there were no differences in reports of W1 internalizing or externalizing behaviors or any other demographic characteristic (age, nativity, maternal educational attainment) for those who remained in the study compared to those who were lost to attrition at W2. As reported in previous studies using these data (e.g., Partovi et al., 2025), there was little missing data due to item non-response with the exception of mothers’ educational attainment at W1, which was missing for 16.1% of participants. Thus, missing data due to survey and item non-response was determined to be MCAR or MAR (missing at random), for both of which FIML (full-information maximum likelihood) is appropriate for handling. Item missing data for maternal educational attainment was multiply imputed using the Multiple Imputation by Chained Equations package in R version 3.4.4 (van Buuren & Groothuis-Oudshoorn, 2011). A single value for maternal education attainment was obtained by pooling the results across all imputed datasets, which allowed us to account for the between-imputation variance that comes with imputed values. Given that maternal education was used as a covariate, we reasoned that this method would have minimal impact on the results (Z. Stickley, personal communication, 28 April 2026).

Data for mentor relationship quality were missing by design for youth who reported not having a mentor. We used dummy variable adjustment (P. D. Allison, 2003; P. Allison, 2022), using residual centering to generate random values uncorrelated with the relationship quality scores to replace the values missing by design (T. Little, personal communication, 19 January 2026). This enabled us to include the measures of both mentor presence and relationship quality simultaneously in the same model, as the method ensures that no cases are lost, all available information is incorporated into the model, and estimated standard errors are accurate (P. Allison, 2022). Moreover, in cases where data are missing because the variable has no meaning (e.g., relationship quality has no meaning for youth with no mentor), this approach also yields unbiased estimates of the coefficients (P. Allison, 2022).

## 3. Results

### 3.1. Study Sample and Descriptive Statistics

Examination of descriptive statistics (See Table 1 for statistics spanning all 11 waves) revealed that girls reported higher discrimination and internalizing problems at W9, on average, compared to boys (M = 0.68, SD = 0.80 and M = 0.50, SD = 0.71, respectively; *p* < 0.05). Correlations among study variables (Table 2) showed that, as expected, girls reported more internalizing problems than boys did (*p* < 0.01) from W2 through W11. Gender differences in externalizing problems were not significant at any study wave. Age was positively related to externalizing problems only at W1 and mothers’ education was negatively related to externalizing problems at W1 and W2. Gender and age were correlated with discrimination at W9, such that girls and older youth reported more discrimination than others. No correlations of mentoring variables with gender, age, mothers’ education, or generation status reached significance. Both externalizing and internalizing problems had significant positive correlations with discrimination across study waves (*r*s ranged from 0.28 to 0.46 for externalizing and from 0.30 to 0.42 for internalizing). Internalizing problems measured at W1–W8 had weak and mostly non-significant negative correlations (between 0 and −0.10) with presence of a mentor. The correlations of mentor presence with concurrent (W9) internalizing problems was somewhat stronger (*r* = −0.17), reverting to weaker negative correlations in W10–W11. Similarly, among youth with a mentor, internalizing problems at W1–W8 had weak negative correlations (between 0.00 and −0.11) with relationship quality, and somewhat stronger negative correlations in W9–W11. Similarly, prior externalizing problems were uncorrelated with W9 mentor presence, and only weakly and negatively correlated with W9 relationship quality. Relationship quality (but not mentor presence) was somewhat more strongly and negatively correlated with externalizing problems in W9–W11. Taken together, these preliminary findings suggest that prior internalizing and externalizing problems played little part in whether young people identified a mentor by W9. The weak, but significant correlations of prior externalizing problems with quality of mentor–mentee relationships suggest that, despite similar likelihood of having a mentor, youth exhibiting externalizing problems may experience less positive relationships than youth with fewer externalizing problems. The stronger correlations of mentoring variables with subsequent internalizing and externalizing problems point to a potential role of mentoring variables as resilience promotive factors.

### 3.2. Longitudinal Trajectories of Internalizing and Externalizing Problems

We estimated latent growth curve models spanning W1 through W11 separately for internalizing and externalizing problems, including both linear and quadratic slopes for each outcome. We began by fitting a series of multigroup models by gender, including both linear and quadratic slopes. We set the intercept at W9 to correspond with the timing of the assessment of mentoring and discrimination and modeled half-point intervals between W1 and W8 and one-point intervals between W9 and W11.

#### 3.2.1. Internalizing Problem Trajectories

**Figure 1:**
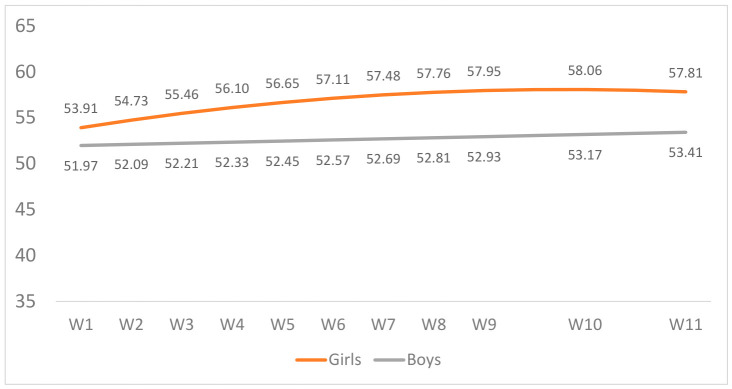
Model-estimated internalizing problems for girls and boys from W1 (Spring 2018) when youth were 11–16 years of age to W11 (Fall 2024).

The initial internalizing model, which allowed the intercepts and both the linear and quadratic slopes to be freely estimated for girls and boys, fit the data well, χ^2^ (117 *d.f.*) = 240.56, *p* = 0.000, CFI = 0.962, RMSEA = 0.064, SRMR = 0.043 (Table 3). A second model, which imposed equality constraints on all three parameters, significantly degraded the model fit, ∆χ^2^ (3 *d.f.*) = 25.90, *p* = 0.000, indicating that gender differences were present. To isolate the source of gender differences, we further tested constraints on each parameter individually and in combination, finding that a model with equality constraint only on the linear slope fit as well as the initial model, ∆χ^2^ (1 *d.f.*) = 0.61, ns. Overall, there was a significant linear increase in internalizing problems over time, M_slope_ = 0.38, *SE* = 0.15, *p* = 0.01, which varied significantly between persons, Var_sres_ = 5.23, *SE* = 0.81, *p* = 0.00. The model for girls included an intercept of 57.98, *SE* = 0.71, *p* = 0.00, which varied significantly between persons, Var_ires_ = 134.19, *SE* = 12.38, *p* = 0.00, and a significant negative quadratic slope, M_quad_ = −0.16, *SE* = 0.05, *p* = 0.00 which also varied significantly between persons, Var_qres_ = 0.38, *SE* = 0.07, *p* = 0.00. For boys, the model included an intercept of 52.72, *SE* = 0.79, *p* = 0.00, which varied significantly between persons, Var_ires_ = 115.97, *SE* = 13.35, *p* = 0.00, and a non-significant quadratic slope. As shown in Figure 1, whereas both girls and boys experienced increased internalizing problems over time, the slope for girls began to taper off by W8 (Fall 2021) when most youth were in grades 9–11 (15 to 19 years old). Girls’ internalizing problems at W1 were slightly above the population mean of T = 50, increasing by nearly half a standard deviation by W11. Boys’ internalizing problems increased by about 1/5 of a standard deviation. Figure 1 illustrates the model-estimated trajectories of internalizing problems for girls and boys from W1 (Spring 2018) when youth were 11–16 years of age to W11 (Fall 2024) when youth were 17–21 years of age. Youth were between 15 and 19 years old at W9 (Fall 2022) when mentoring and discrimination measures were assessed.

#### 3.2.2. Externalizing Problem Trajectories

We estimated models for externalizing problems similarly, except that we omitted the quadratic slopes, which did not reach significance for either girls or boys. The initial model allowed the intercepts and the linear slope to be freely estimated across gender and fit the data adequately, χ^2^ (122 *d.f.*) = 333.59, *p* = 0.000, CFI = 0.936, RMSEA = 0.080, SRMR = 0.062. The second model, which imposed equality constraints on the intercept and slope, fit about as well as the unconstrained model, ∆χ^2^ (2 *d.f*.) = 2.14, ns, indicating that girls and boys shared similar levels of externalizing problems and a similar pattern of increasing externalizing problems over time. The mean at the intercept was 49.74 (*SE* = 0.42), *p* = 0.00, and varied significantly between persons, Var_ires_ = 92.40, *SE* = 10.36, *p* = 0.00. On average externalizing problems increased linearly over time M_slope_ = 0.26, *SE* = 0.09, *p* = 0.00 and varied significantly between persons, Var_sres_ = 2.09, *SE* = 0.43, *p* = 0.00. Externalizing problems increased by about half of a standard deviation from a mean of 45.10 at W1 to a mean of 51.04 at W11.

### 3.3. Explanatory Models of Discrimination and Mentoring

#### 3.3.1. Internalizing Problems

Given gender differences in the longitudinal trajectories of internalizing problems, we estimated subsequent explanatory models separately for girls and boys. The explanatory model for girls (See Table 4) fit the data well, χ^2^ (136 *d.f*.) = 234.086, CFI = 0.958; RMSEA = 0.049; SRMR = 0.050. The model explained 25% of the variance in the intercept, 16% of the variance in the linear slope, and 15% of the variance in the quadratic slope. The only covariate contribution was a significant and positive effect of age on the quadratic slope, suggesting that the slope accelerated for older girls over time. Mentor presence was associated with fewer internalizing problems at the intercept, consistent with the compensatory resilience model; furthermore, a significant association of mentor presence with the quadratic slope suggests that this association weakened over time. For girls with a mentor, relationship quality moderated both the intercept and the linear slope of the association between discrimination and internalizing problems.

**Figure 2:**
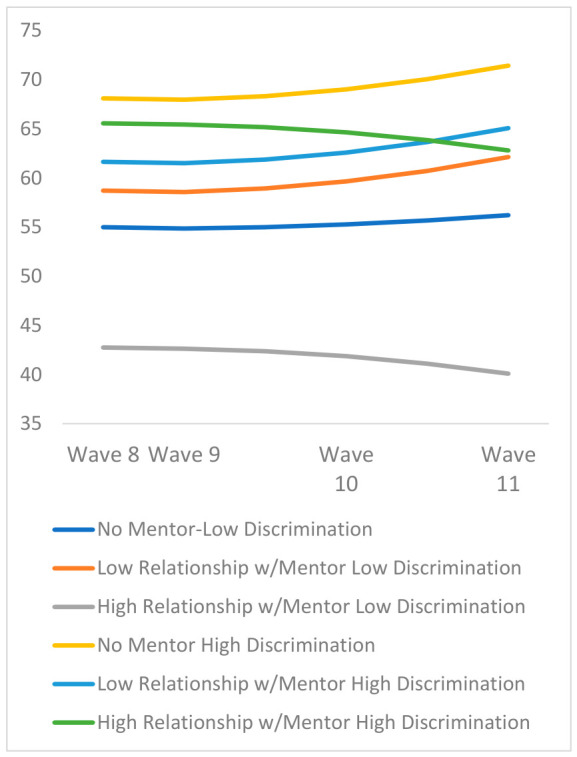
Model-implied trajectories from W8 to W11 of girls’ internalizing problems.

To understand these interactions, we computed simple slopes comparing girls with positive mentoring relationships (i.e., 1 unit above the mean) to those with less positive relationships (i.e., 1 unit below the mean). For girls who reported less positive relationships, discrimination was associated with more internalizing problems, *b* = 7.54, *SE* = 1.84, *p* < 0.01, and with a steeper linear increase over time, *b* = 1.04, *SE* = 0.47, *p* < 0.05. In contrast, girls who reported more positive relationships with their mentors did not experience significantly more internalizing problems at the intercept, *b* = 3.13, *SE* = 2.00, *ns*, and showed a relative decline in the slope over time, *b* = −1.01, *SE* = 0.52, *p* = 0.05. Figure 2 illustrates the model-implied trajectories of girls’ internalizing problems for girls with no mentors, and girls who reported low and high relationship quality at low and high levels of discrimination. Evidenced by the declining slopes over time, support for the risk-protective model implied by the simple slopes analyses appears to hold only for girls who reported positive relationships with their mentors.

The explanatory model for boys (See Table 5) showed adequate fit to the data, χ^2^ (147 *d.f.*) = 235.798, CFI = 0.923; RMSEA = 0.050; SRMR = 0.095. The model explained 17% of the variance in the intercept, and 16% of the variance in the linear slope. Older boys reported more internalizing problems at the intercept than their younger peers, and U.S.-born youth showed less steep increases over time. Mentor presence was unrelated to either the intercept or slope. However, among boys with a mentor, those with more positive relationship quality reported fewer internalizing problems at the intercept. The interaction of discrimination X relationship quality accounted significantly for variance in the slope of internalizing; however, the simple slopes were not significant for either positive (1 unit above the mean), *b* = −0.35, *SE* = 0.82, *ns*, or less positive (1 unit below the mean), *b* = 0.95, *SE* = 0.89, *ns*, relationship quality. Thus, this analysis supports the compensatory model for boys internalizing problems.^2^

#### 3.3.2. Externalizing Problems

**Figure 3:**
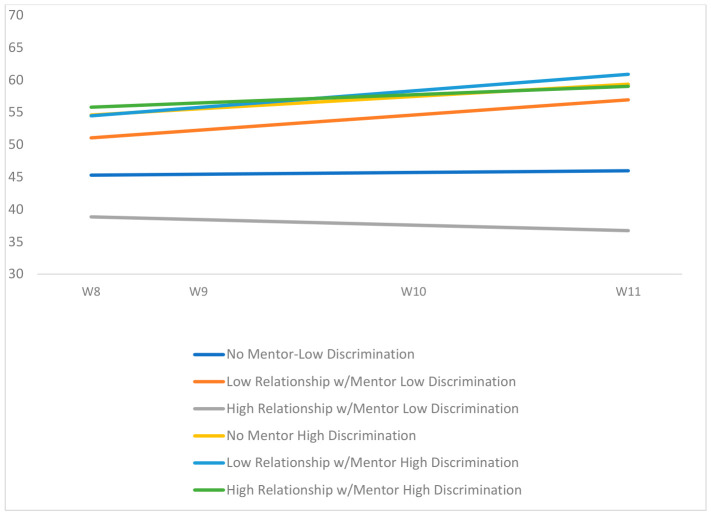
Model-implied trajectories from W8 to W11 of externalizing problems.

Given that the multigroup model for externalizing problems revealed no gender differences in the intercept or slope, we estimated the explanatory model with the combined sample, adding gender as a covariate. The model (See Table 6) fit the data adequately, χ^2^ (162 *d.f*.) = 404.833, CFI = 0.929; RMSEA = 0.052; SRMR = 0.071. The model explained 27% of the variance in the intercept and 18% of the variance in the linear slope. Externalizing problems increased less steeply for U.S.-born youth compared to those born outside the U.S. and more steeply for youth with more highly educated mothers. Mentor presence moderated the association between discrimination and the slope of externalizing problems. Inspection of the simple slopes revealed that, youth who did not have a mentor reported greater increases in externalizing problems over time, *b* = 8.45, *SE* = 1.62, *p* < 0.001, whereas those with a mentor did not, *b* = 1.67, *SE* = 1.73, *ns*. Among youth with a mentor, relationship quality moderated both the intercept and slope of externalizing. Inspection of the simple slopes revealed non-significant associations at the W9 intercept between discrimination and both the intercept, *b* = 0.59, *SE =* 0.33, *ns*, and slope, *b* = 0.18, *SE =* 0.38, *ns*, of externalizing problems for youth reporting a positive relationship with their mentors, but significant associations of discrimination with both the intercept, *b* = 1.05, *SE =* 0.33, *p* < 0.001, and slope, *b* = 1.47, *SE =* 0.35, *p* < 0.001, of externalizing for youth reporting a relatively poor relationship with their mentors. Figure 3 illustrates the model-implied trajectories from W8 through W11 at low (1 unit below the mean) and high (1 unit above the mean) levels of discrimination for youth with no mentor, and youth with low (1 unit below the mean) and high (1 unit above the mean) levels of relationship quality.

## 4. Discussion

Framed within resilience theory, the current study of Latino/a late adolescent girls and boys investigated informal mentoring as a resilience factor in the association of discrimination with longitudinal trajectories of internalizing and externalizing problems. This study added to the limited research on mentoring in the Latino/a population by investigating processes contributing to behavioral health over time and by examining both mentor presence and relationship quality. We considered two primary resilience models: compensatory model in which positive main effects of mentoring directly offset negative effects of discrimination, and a risk-protective model, in which mentoring moderated, or buffered, the effects of discrimination. Results were largely consistent with prior findings from studies of Black or diverse samples (DuBois & Silverthorn, 2005; Hurd & Zimmerman, 2010a, 2010b; Kogan et al., 2011; Rhodes et al., 1994), and provided evidence for both resilience models, depending on gender and the outcome examined.

For girls, whereas the direct association of mentor presence with internalizing problems at the intercept was consistent with the compensatory resilience model, and interaction effects of relationship quality X discrimination with both the intercept and the linear slope suggested support for the risk-protective model, inspection of the simple slopes and the model-implied trajectories suggested a more nuanced interpretation. That is, declining levels of internalizing problems W9 to W11 implied by simple slopes analysis appeared to characterize only those girls who reported a positive relationship with their mentors. Girls without mentors and those with less positive mentor–mentee relationships appeared to experience stable or increasing levels. Notably, girls who reported high levels of discrimination experienced markedly more internalizing problems over time, with averages bordering or exceeding clinically significant levels (i.e., T-scores ≥ 64, Achenbach & Edelbrock, 1991).

For boys, the direct effect of mentor relationship quality at the intercept reached significance, suggesting that positive relationships with mentors were associated with lower levels of internalizing problems. A weak but significant relationship quality X discrimination interaction on the slope of internalizing could not be interpreted as the simple slopes for the association between discrimination and internalizing problems failed to reach significance at either low or high relationship quality. Because the zero order correlation between discrimination and internalizing problems was significant, the beneficial association between mentor relationship quality and internalizing problems at the intercept could be interpreted as consistent with the compensatory model.

Analysis of externalizing problems was carried out using the combined sample. Consistent with the risk-protective model, mentor presence moderated the association of discrimination with the slope of internalizing and, among youth with a mentor, relationship quality moderated the association of discrimination with both the intercept and slope. Inspection of the simple slopes and model-implied trajectories suggested that the risk-protective effect of a positive mentor–mentee relationship might accrue only to youth experiencing relatively low levels of discrimination. The pattern of the trajectories implied by the model suggests that high levels of exposure to discrimination overwhelm the potential resilience-promoting effects of mentoring on externalizing problems.

Whereas our analyses presuppose a direction of effects from mentor presence and mentor relationship quality to behavioral health, the possibility of bidirectional effects cannot be ruled out. As noted previously, it is also possible that unmeasured variables that affected both behavioral health and mentoring might account for the observed effects. Resilience is best viewed as a dynamic process involving the co-action of individual attributes and contextual resources and risks that themselves are changing over time; thus, bidirectional influences are to be expected (Lerner et al., 2023). Indeed, both internalizing and externalizing problems in W1 through W8 of the study had substantial correlations (ranging from 0.28 to 0.43) with reports of discrimination at W9. The weak and mostly nonsignificant correlations of W1–W8 internalizing and externalizing problems and W9 mentor presence suggest that behavioral health difficulties were not a major selection factor for having a mentor. However, the small but statistically significant associations of earlier externalizing problems with relationship quality suggest that youth who act out their distress through rule breaking or aggressive behavior might struggle to establish positive mentoring relationships. Such a pattern is consistent with the findings of Sánchez et al. (2017), whose study of risk behavior trajectories in a sample of Latino/a high school students showed that mentoring relationship quality decreased as discrimination increased over time. Relatedly, Stanton-Salazar and Spina’s (2003) ethnographic study of the social networks of Mexican American youth from immigrant families highlighted a range of barriers to securing mentors, including challenges related to cultural assimilation, segregation, resource-poor schools, economic disadvantage, and community violence. Despite such challenges, all of which are likely to increase risk for emotional and behavioral adjustment difficulties, some youth in that study were able to form and maintain strong and long-lasting relationships with a mentor. The authors noted that even when mentoring relationships lacked long-term stability, they could nevertheless be important at key moments, for example, when youth were facing particularly difficult moments in their lives. These findings underscore the multi-layered, ecological influences that shape mentor access for Latino/a youth. Thus, while the current study’s findings provide support for viewing mentoring as a resilience factor, it is clear that the contributions of mentoring to youths’ wellbeing must be considered within a broader ecological context (Zimmerman et al., 2013).

### 4.1. Limitations and Future Directions

This study examined gender differences using gender reported at baseline; therefore, findings might not generalize to adolescents whose gender identities change over time. Additionally, the COVID-19 pandemic, which coincided with earlier waves of this study, may have influenced internalizing and externalizing trajectories. Prior research among Latino/a adolescents, including the current sample, shows higher internalizing and externalizing problems post-onset compared to pre-pandemic levels (Polo et al., 2024; Roche et al., 2022). The statistical models explained moderate proportions of variance in the intercepts (ranging from 17% to 29%) and slopes (ranging from 15% to 18%) of externalizing and externalizing problems; thus, whereas the findings support a role of informal mentoring in promoting resilience among Latino/a youth, other factors may be equally or more important, including external resources such as parental or peer support, as well as internal assets, such as a positive racial/ethnic identity and coping skills.

We were able to capitalize on data from a long-term longitudinal study with multiple observations to estimate seven-year trajectories of internalizing and externalizing problems. However, the introduction of mentoring measures and a new measure of everyday discrimination only at W9 raised challenges to interpreting the findings. Having multiple earlier waves of data on the sample provided robust information about ‘where they came from,’ how mentoring and discrimination were associated concurrently at the time those measures were added to the study, and how mentoring and discrimination may have contributed to where ‘they were going’ with regard to behavioral health. It is important to recognize that, although consistent with hypothesized resilience models, concurrent associations at W9 might also reflect effects of mentoring at prior waves (before the study assessed it) or of third variables that affected behavioral health, mentoring, and exposure to discrimination. The observational design of the study restricted our ability to infer causation. However, our approach of simultaneously examining mentor presence and, relationship quality created a quasi-counterfactual, enabling us to examine how longitudinal trajectories might differ in subsequent study waves for youth varying in exposure to discrimination and differing in whether they identified a mentor and, among those with a mentor, the quality of the mentor–mentee relationship.

Reliance on self-report for all of the study measures raises the likelihood that estimates were biased due to shared method variance. Although assessed with a single item, mentor presence was clearly defined using a commonly accepted operationalization, and relationship quality was measured with a well-validated and widely used measure that captured both instrumental and relational quality. Future research should examine potential distinct effects of these dimensions of relationship quality.

Mentoring measures were not introduced into the study until W9, and additional details, such as mentor characteristics, frequency of contact, duration and stability of the relationship over time were not available. We had no way of knowing whether mentoring relationships endured over multiple waves, or whether differences in assessment of relationship quality from one wave to the next reflected change over time or a relationship with a different mentor. The lack of such detail made it difficult to test models that could fully examine longitudinal and bidirectional effects. However, because few studies have examined how informal mentors can contribute to behavioral health among Latino/a youth from immigrant families, the current study offers a baseline for future research that can address more nuanced questions regarding mentoring and resilience processes.

#### 4.1.1. More Nuanced Examination of Mentoring Processes

Greater detail about mentors and relationship processes would allow for more refined analysis, such as comparing effects of long-term mentor–mentee relationships, with less stable relationships. Past research has noted that minoritized youth are often more likely than their peers to recruit mentors from their proximal networks, including extended family members, family friends, and neighbors (Gowdy et al., 2020; Putnam, 2015). From a social capital perspective, Gowdy and Spencer (2021) identified these as “core” mentors, noting that they may be best equipped to provide emotional support and reassurance because they often share a similar background and have greater knowledge of the youth and their family. At the same time, core mentors might struggle with challenges (e.g., discrimination) similar to those of their mentees. Consequently, opportunities for such mentors to help young people deal with discrimination might be lost due to lacking confidence to raise difficult conversations with their mentees or waiting for the mentee to bring them up (Sánchez et al., 2026). Young people may become dissatisfied with the relationship if they feel that their mentors are not helping them deal with discrimination or other important issues in their lives, perhaps exacerbating the negative impacts of those experiences.

Gowdy and Spencer (2021) differentiated core mentors from “capital” mentors, that is, those recruited from more distal parts of youths’ social networks (e.g., teachers, coaches); such mentors may be better equipped to provide informational support and link youth to resources. However, because such mentors often do not share their mentee’s racial, geographic, or socioeconomic backgrounds, it may take longer to establish the level of trust and connection needed to address emotionally charged experience (Sánchez et al., 2026). It is important that future research consider how and why young people seek out mentors, the types of support that mentors are able to provide, and whether and how forging relationships with multiple mentors affords young people additional advantages.

#### 4.1.2. Expanded Examination of Resilience Processes

This study focused on the two most frequently studied resilience models: the compensatory and risk-protective models. For example, consideration of a protective–protective model might consider processes through which mentoring or other resources external to the individual integrate with youths’ internal assets to affect youths’ adjustment in the face of discrimination. Informal mentors have been found to support the development of cultural identity (De Los Reyes et al., 2022), coping efficacy (Sánchez et al., 2020), and self-regulation skills (Kogan et al., 2011), all of which have been shown to be related to better behavioral health.

Noting that research on mentoring racially and ethnically marginalized youth has lacked a specific theoretical framework, Hurd (2024) has advanced a model aligned with the positive youth development framework (e.g., the five C’s of competence, confidence, character, caring, connection, and contribution; Geldhof et al., 2015) that emphasizes the mentor’s role in supporting a young person’s racial and cultural socialization, and development of critical reflection, skill building, advocacy, and support for coping as pathways toward active engagement in combatting racism. Hurd’s model points to possibilities for a less frequently studied resilience model, the challenge model, which considers ways that risk exposure can actually help youth overcome further exposure (Zimmerman et al., 2013). For example, Delbasso et al.’s (2025) study of immigrant-origin emerging adults of color found that interpersonal discrimination and concerns about structural discrimination (e.g., anti-immigrant policies) were linked to higher levels of civic engagement. Structural discrimination concerns predicted social activism among youth who reported high levels of ethnic/racial identity exploration. Given that past research has found associations of mentoring both with identity development and civic engagement (De Los Reyes et al., 2022), future research can consider ways that mentoring can help to promote growth through challenging experience, not just in spite of it.

### 4.2. Implications and Conclusions

Whereas most youth in the current study reported having at least one mentor, past research has found considerable variability in minoritized youth’s ability to access reliable support from adults or older peers outside of their immediate families. For example, whereas the staff in afterschool programs offer a ready pool of adults eager to mentor youth in their care (Hirsch et al., 2011), Latino/a youth are often under-represented in them, either because programs are unavailable in their communities or because they perceive programs as lacking cultural responsiveness (Riggs et al., 2010). Youth-initiated mentoring is an innovative and promising approach that combines the strengths of informal mentoring with supports provided by formal programs by training youth to identify and recruit natural mentors (van Dam et al., 2020). Supports to encourage potential mentors to engage with youths and, once engaged, to become effective mentors are also needed. For example, workplace policies that reward employees for mentoring youth either informally or through partnerships with schools or formal mentoring programs (Garringer et al., 2017) can play an important role in increasing the pool of mentors, particularly in communities that serve large proportions of Latino/a and other underserved groups. Such partnerships can also serve as hubs for providing mentor training and peer support.

Discrimination consistently shows harmful effects on Latino/a adolescents’ emotional and behavioral adjustment. Within this context, a resilience perspective underscores the reality that most youth are able to overcome those risks with adequate support. The current study fills gaps in the current literature by examining how informal mentoring can play a role in fostering resilience to such harmful effects, and highlights directions for future research and practice aimed at enhancing the well-being of this large and fast-growing population.

## Figures and Tables

**Table 1:** Descriptive statistics for study variables by gender (*N* = 547) ^a^.

	Overall M (SD) or ƒ (%)	Boys M (SD) or ƒ (%)	Girls M (SD) or ƒ (%)	χ^2^ or *t*
Age (W1)	13.31 (0.97)	13.29 (1.02)	13.32 (0.93)	0.26 (545 *d.f.*)
Gender				--
Girls	303 (55.4%)	--	--	
Boys ^b^	244 (44.6%)	--	--	
U.S.-Born	490 (89.6%)	219 (89.8%)	271 (89.4%)	0.01 (1 *d.f.*)
Mother’s Education				6.73 (5 *d.f.*)
Less than HS graduate	216 (39.3%)	91 (37.3%)	124 (40.9%)	
HS graduate	126 (23.0%)	56 (23.0%)	70 (23.1%)	
Some college	47 (8.6%)	23 (9.4%)	24 (7.9%)	
Completed college	115 (21.0%)	58 (24.2%)	56 (18.5%)	
Graduate/Professional School	44 (8.0%)	15 (6.1%)	29 (9.6%)	
Household Structure ^c^				0.76 (2 *d.f.*) ^d^
Two biological/adoptive parents	367 (67.3%)	161 (66.3%)	206 (68.2%)	
Stepparent in household	73 (13.4%)	36 (14.8%)	37 (12.3%)	
Single parent	99 (18.1%)	43 (17.6%)	56 (18.5%)	
No parent	6 (1.1%)	3 (1.2%)	3 (1%)	
Presence of Mentor (W9)				1.26 (1 *d.f.*)
Yes	267 (72.2%)	105 (75.5%)	162 (70.1%)	
No	103 (27.8%)	34 (24.5%)	69 (29.9%)	
Mentor Relational Quality (W9)	3.38 (0.53)	3.37 (0.53)	3.39 (0.53)	0.28 (265 *d.f.*)
Discrimination (W9)	0.61 (0.78)	0.50 (0.71)	0.68 (0.80)	2.23 (367 *d.f.*) *
Internalizing Problems (W9)	57.29 (13.32)	54.96 (13.23)	58.71 (13.20)	2.64 (363 *d.f.*) **
Externalizing Problems (W9)	50.02 (10.90)	49.66 (11.54)	50.24 (10.51)	0.49 (363 *d.f.*)

*Note.* ^a^. Descriptive statistics for demographic variables were computed using the full sample at baseline (*N* = 547); descriptive statistics for mentoring, discrimination, and internalizing and externalizing symptoms were computed using the subset of participants with complete data at the respective wave. ^b^. *N* = 3 boys were not included in the final analytic models due to incomplete data for internalizing and externalizing symptoms across all waves. ^c^. numbers may not sum to total sample size due to item-level missing data. ^d^. Due to the small number of no-parent households, this category was combined with single-parent households for the chi-square test. W1 = Wave 1; W9 = Wave 9; *d.f.* = degrees of freedom; HS = high school. ** *p* < 0.01; * *p* < 0.05.

**Table 2:** Bivariate correlations (*N* = 544).

	1	2	3	4	5	6	7	8	9	10	11	12	13	14	15	16	17	18
1. W9 Age	--	−0.11	−0.12	0.02	0.09	0.14	−0.08	0.16	0.12	0.06	0.10	0.05	0.06	0.04	0.05	0.01	0.10	0.09
2. U.S.-Born	−0.11	--	−0.01	0.00	−0.07	−0.05	0.11	0.07	0.03	0.03	−0.05	−0.05	0.02	0.00	0.00	−0.02	−0.04	−0.07
3. Mother Ed	−0.12	−0.01	--	0.04	0.04	−0.05	0.04	−0.08	−0.09	−0.01	0.01	0.03	0.03	−0.01	0.06	0.04	0.02	0.04
4. Gender (1 = boys)	0.02	0.00	0.04	--	−0.03	−0.01	−0.07	−0.01	0.00	0.04	−0.02	0.04	−0.04	−0.05	−0.08	−0.01	−0.04	−0.03
5. Discrimination	0.13	0.04	0.06	−0.11	--	0.06	−0.01	0.32	0.28	0.39	0.35	0.30	0.43	0.42	0.36	0.46	0.42	0.35
6. Mentor Presence	−0.06	−0.04	−0.01	0.05	−0.01	--	--	0.07	0.04	0.03	0.04	0.04	0.04	−0.05	−0.00	−0.02	−0.00	0.03
7. Mentor Rel.	−0.08	0.12	0.04	−0.07	−0.08	--	--	−0.01	−0.13	−0.11	−0.09	−0.10	−0.10	−0.10	−0.11	−0.11	−0.19	−0.21
8. W1 Ext or Int	0.11	0.03	−0.07	−0.06	0.30	−0.02	0.01	--	0.69	0.57	0.56	0.50	0.52	0.48	0.51	0.45	0.44	0.42
9. W2 Ext or Int	0.12	0.05	−0.01	−0.12	0.32	−0.01	−0.09	0.69	--	0.74	0.68	0.60	0.61	0.59	0.56	0.51	0.51	0.47
10. W3 Ext or Int	0.06	−0.02	0.02	−0.09	0.35	−0.06	−0.09	0.60	0.73	--	0.74	0.67	0.73	0.66	0.61	0.64	0.56	0.53
11. W4 Ext or Int	0.11	−0.03	0.05	−0.15	0.35	−0.06	−0.08	0.62	0.70	0.74	--	0.72	0.72	0.67	0.61	0.59	0.55	0.51
12. W5 Ext or Int	0.12	−0.03	0.01	−0.14	0.30	−0.04	−0.09	0.53	0.59	0.66	0.75	--	0.70	0.65	0.63	0.64	0.52	0.51
13. W6 Ext or Int	0.11	0.02	−0.02	−0.18	0.42	−0.07	−0.11	0.54	0.63	0.67	0.69	0.70	--	0.76	0.73	0.70	0.59	0.58
14. W7 Ext or Int	0.03	0.00	0.01	−0.22	0.36	−0.10	−0.03	0.51	0.60	0.60	0.66	0.66	0.74	--	0.76	0.76	0.64	0.60
15. W8 Ext or Int	0.09	−0.02	0.02	−0.24	0.35	−0.10	−0.11	0.49	0.57	0.57	0.64	0.63	0.71	0.76	--	0.76	0.61	0.64
16. W9 Ext or Int	0.06	−0.04	−0.02	−0.14	0.42	−0.17	−0.11	0.47	0.50	0.55	0.59	0.61	0.68	0.69	0.73	--	0.67	0.68
17. W10 Ext or Int	0.07	−0.05	0.01	−0.13	0.36	−0.09	−0.19	0.43	0.48	0.48	0.52	0.54	0.56	0.61	0.62	0.70	--	0.69
18. W11 Ext or Int	0.10	−0.08	0.03	−0.13	0.35	−0.06	0.22	0.39	0.46	0.48	0.53	0.58	0.56	0.61	0.64	0.66	0.75	--
Mean (SD)	17.46 (0.93)	0.90 (0.30)	3.14 (1.63)	0.45 (0.50)	0.61 (0.77)	0.72 (0.45)	3.39 (0.55)	48.73 (10.25)	48.64 (10.13)	48.44 (10.91)	48.00 (10.55)	49.32 (10.57)	48.72 (10.50)	49.09 (10.35)	48.59 (10.28)	50.02 (10.88)	49.94 (10.73)	50.23 (11.68)
								53.85 (11.91)	53.45 (12.56)	53.41 (13.04)	53.50 (13.15)	54.88 (13.16)	54.52 (13.71)	55.33 (13.21)	55.85 (13.41)	57.29 (13.30)	55.96 (13.69)	56.60 (13.91)

Note: Estimated correlations from final structural equation models for externalizing (above diagonal) and internalizing (below diagonal) problems. Means were estimated in *Mplus* using full information maximum likelihood to account for missing data. Externalizing means are presented in the top row; internalizing means are presented in the bottom row. Int = internalizing symptoms; Ext = externalizing symptoms; W = wave; Rel = relational quality; SD = standard deviation. Correlations with absolute values above 0.08 are significantly different from 0 with *p* < 0.05.

**Table 3:** Fit statistics for multiple group latent growth curve models examining trajectories of internalizing and externalizing problems (*N* = 544).

	χ^2^ (*d.f.*)	∆χ^2^ (*d.f.*)	RMSEA	CFI	SRMR
Internalizing Problems					
No equality constraints	240.56 (114)	---	0.064	0.962	0.043
Constrained intercept, linear, and quadratic slopes	266.46 (117)	25.90 ** (3)	0.069	0.955	0.068
Constrained linear slope only	241.17 (115)	0.61 *ns* (1)	0.064	0.962	0.043
Externalizing Problems					
No equality constraints	333.59 (122)	---	0.080	0.936	0.062
Constrained intercept, linear, and quadratic slopes	335.73 (124)	2.14 (2)	0.079	0.936	0.062

*Note*: ** *p* < 0.01.

**Table 4:** Latent growth curve model examining Wave 9 discrimination and mentoring on girls’ internalizing problems (*N* = 303).

	Intercept		Linear Slope		Quadratic Slope	
	*b*	*SE*	*b*	*SE*	*b*	*SE*
Intercept	61.42 ***	2.28	0.77	0.62	−0.32 †	0.19
Age	−0.06	0.72	0.1	0.2	0.12 *	0.06
U.S.-Born	−0.20	2.1	−0.46	0.57	−0.10	0.17
Mother’s Education	0.21	0.38	0.02	0.11	−0.03	0.03
Discrimination	6.56 ***	1.67	−0.03	0.44	−0.27 *	0.14
Mentor Presence	−4.28 **	1.5	0.01	0.4	0.34 **	0.12
Mentor Relationship Quality	−3.01	1.87	−1.40 **	0.48	−0.13	0.15
Discrimination X Mentor Presence	−0.02	2.01	−0.29	0.53	0.21	0.16
Discrimination X Mentor Relationship Quality	4.97 *	2.36	2.43 ***	0.62	0.13	0.19
Residual Variance	94.55 ***	9.4	4.36 ***	0.75	0.30 ***	0.07
R-Squared	0.29 ***		0.15 **		0.16 *	

Note: *** *p* < 0.001; ** *p* < 0.01; * *p* < 0.05; † *p* < 0.10. Unstandardized estimates are shown. Model fit: χ^2^ (136) = 234.086; RMSEA = 0.049; CFI = 0.958; SRMR = 0.050.

**Table 5:** Latent growth curve model examining Wave 9 discrimination and mentoring on internalizing problems for boys (*N* = 241).

	Intercept		Linear Slope	
	*b*	*SE*	*b*	*SE*
Intercept	54.18 ***	2.94	2.89 ***	0.56
Age	1.64	0.82	0.13	0.19
U.S.-Born	−2.54	0.24	−1.25 *	0.58
Mother’s Education	0.04	0.46	0.1	0.1
Discrimination	4.46	3.19	0.3	0.67
Mentor Presence	1.48	2.12	0.41	0.45
Mentor Relationship Quality	−5.84 *	2.48	−0.65	0.54
Discrimination X Mentor Presence	−0.46	3.51	−0.07	0.74
Discrimination X Mentor Relationship Quality	5.58	3.88	1.78 *	0.84
Residual Variance	90.18 ***	11.14	2.89 ***	0.56
R-Squared	0.17 **	0.06	0.16 *	0.07

*Note*: *** *p* < 0.001; ** *p* < 0.01; * *p* < 0.05. Unstandardized estimates are shown. Model fit: χ^2^ (162) = 235.798; RMSEA = 0.050; CFI = 0.923; SRMR = 0.095.

**Table 6:** Latent growth curve models examining Wave 9 discrimination and mentoring on externalizing problems (*N* = 544).

	Intercept		Linear Slope	
	*b*	*SE*	*b*	*SE*
Intercept	50.38 ***	1.46	1.09 ***	0.32
Age	0.21	0.43	−0.14	0.1
U.S.-Born	−0.90	1.28	−0.61 *	0.29
Mother’s Education	0.16	0.23	0.14 **	0.05
Sex (0 = female; 1 = male)	−0.33	0.78	−0.24	0.17
Discrimination	5.06 ***	1.17	0.82 ***	0.25
Mentor Presence	0.44	0.97	−0.23	0.21
Mentor Relationship Quality	−3.39 **	1.2	−0.64 *	0.26
Discrimination X Mentor Presence	0.43	1.35	−0.71 *	0.29
Discrimination X Mentor Relationship Quality	3.52 *	1.57	0.96 **	0.34
Residual Variance	59.80 ***	4.72	1.76 ***	0.23
R-Squared	0.27 ***	0.04	0.18 ***	0.05

*Note*: * *p* < 0.05., ** *p* < 0.01; *** *p* < 0.001. Unstandardized estimates are shown. Model fit: χ^2^ (162) = 404.833; RMSEA = 0.052; CFI = 0.929; SRMR = 0.071.

## Data Availability

Data can be made available upon request to the study PI, Kathleen Roche (kroche@gwu.edu). Data are not available publicly due to limitations tied to informed consent procedures.

## Notes

1 Although adolescents may use different terms to identify their ethnicity, we use the term Latino/a in this study.

2 A separate examination (not shown above) confirmed that the zero-order correlations of discrimination and internalizing problems were significant across waves for boys, ranging from 0.30 to 0.42.
